# Supplementary material for: Are carbon emissions trading and green financial instruments synergistic? -Comprehensive quantitative research based on content analysis
Source: PLoS One. 2024 Mar 7;19(3):e0298601. doi: 10.1371/journal.pone.0298601 (PMC10919601; doi:10.1371/journal.pone.0298601)
Supplement: S1 Data — (ZIP) [file pone.0298601.s001.zip › Data/地级市Z_F工作报告.zip!/║╙─╧/║╫▒┌╩╨/└┤╫╘╣1⁄2╓┌║┼í╛┬φ┐╦╩2╛▌═°í┐_2018.docx]

各位代表：

　　现在，我代表市人民政府，向大会报告工作，请予审议，并请各位政协委员和其他列席人士提出意见。

　　一、2014年以来工作回顾

　　2014年以来，面对严峻复杂的外部形势和艰巨繁重的工作任务，在市委的正确领导下，市人民政府与全市人民一道，坚持以习近平新时代中国特色社会主义思想为指导，深入贯彻习近平总书记调研指导河南工作时的重要讲话精神，全面落实省委、省政府各项决策部署，解决了许多长期想解决而没有解决的难题，办成了许多过去想办而没有办成的大事，转型发展迈出实质性步伐，改革开放创新取得重大突破，生态文明建设成果丰硕，人民幸福水平不断提高，整体工作保持良好态势，较好完成了市十届人大确定的各项目标任务，为率先全面小康、建设品质“三城”奠定了坚实基础。重点抓了以下工作：

　　（一）着力担当作为，重点攻坚成效显著。坚持把脱贫攻坚作为头等大事和第一民生工程，深入贯彻精准扶贫精准脱贫基本方略，截至2017年底，103个贫困村已脱贫摘帽73个，建档立卡贫困户19164户、57668人，已稳定脱贫11718户、37966人，贫困发生率降至1.82%、低于全省0.75个百分点；今年以来，多项扶贫工作走在全省前列，在省产业扶贫现场观摩活动中被评为豫北五市第一名，省半年重点工作核查被评为“好”的等次。坚持把污染防治攻坚作为重大政治任务和民生工程，深入实施依法治污、科学治污、全民治污，2017年空气优良天数和细颗粒物（PM2.5）平均浓度在省7个通道城市中排名第一，万元工业增加值能耗年均下降7.91%；今年以来，空气质量改善幅度保持省通道城市前列，中央环保督察“回头看”反馈问题全部办结；淇河水质在全省60条城市河流中连年排名第一，地表水责任目标断面达到省定要求，集中式饮用水源地水质达标率保持100%。深入推进转型发展攻坚，坚持以提高发展质量和效益为中心，以推进供给侧结构性改革为主线，以高端化、绿色化、智能化、融合化为方向，加快调整产能、产业、产品结构，截至2017年底，煤炭行业占工业比重下降到6.1%，高新技术产业占规模以上工业增加值的比重提高到31.6%，服务业占生产总值的比重提高到28.2%，资源型城市转型发展迈出实质性步伐，高质量发展实现良好开端。

　　（二）着力稳定增长，经济实力大幅提升。坚持贯彻新发展理念，主动适应把握引领经济发展新常态，强力推进开放招商、项目建设、企业服务工作，经济保持平稳健康增长。2014—2017年，开放招商、项目建设连年保持省先进位次，累计落地亿元以上项目500个、利用省外资金1106亿元、利用境外资金30.7亿美元、完成重点项目投资1563亿元、新建成项目144个，在省项目观摩点评系列活动中，取得豫北片区1次第一名、3次第二名的好成绩，海关、出入境检验检疫局建成投用，产业集聚区、商务中心区实现星级全覆盖；通过强化企业服务，深化体制机制改革，服务实体经济发展能力和营商环境得到进一步提高和优化，市场主体增长102%。2017年全市地区生产总值、规模以上工业增加值、第三产业增加值、固定资产投资、社会消费品零售总额、一般公共预算收入，分别为832.6亿元、454亿元、234.3亿元、901.7亿元、230.5亿元、59.7亿元，年均分别增长8.6%、9.2%、9.2%、16.6%、12.3%、10.8%。今年以来，全市经济运行总体平稳、稳中有进，发展方式转变、经济结构优化、增长动力转换呈现更加积极的变化。

　　（三）着力产业转型，质量效益稳步提高。坚持质量第一、效益优先，努力提高供给体系质量和全要素生产率，加快构建现代产业新体系，经济创新力和竞争力得到持续增强。工业主导地位更加突出，一批百千亿级产业集群快速发展壮大，截至2017年底，清洁能源与新材料、绿色食品、汽车零部件与电子电器、镁精深加工、现代家居5个产业增加值占规模以上工业的96.5%，占生产总值的52.6%，4个省级产业集聚区主营业务收入占规模以上工业的63.3%。农业比较优势持续彰显，粮食总产稳定在110万吨以上，优质小麦、花生、草畜、林果等种植规模稳步扩大，现代农业产业体系、生产体系、经营体系持续完善，农村一二三产融合发展态势更加明显。新型物流、休闲旅游、网络经济、文化创意、健康养老等特色服务业快速发展，带动第三产业呈现速度加快、业态丰富、效益提升、占比提高的良好态势，与2013年相比，2017年第三产业占比提高9.7个百分点。

　　（四）着力城乡建设，承载能力持续增强。坚持以人为核心，积极稳妥推进新型城镇化和城乡一体化，加快构建现代城乡新体系。“一核双星多支点”单元组合型城镇化格局日益完善，主城区、老城区、两县县城、重点乡镇面貌和功能持续改善，城乡一体化示范区、示范镇、示范村等建设体系更加完备，成功创建一批省级以上重点镇、美丽乡村、传统村落等。人居环境持续改善，绿色城市、低碳城市、循环城市、海绵城市、节约城市建设深入推进，森林覆盖率、城市绿地率、城市绿化覆盖率分别达到32.6%、35.66%、39.91%，农村人居环境达标村达到65%。人口转移步伐加快，稳步推进城镇基本公共服务常住人口全覆盖，年均转户落户城镇4万人左右，常住人口城镇化率58.76%、居全省第3位。公共设施更加完善，规划修编、百城提质、城市管理、智慧城市等建设取得新的进展，综合交通体系更加便捷高效，公共用水、燃气、集中供热普及率分别达到96.97%、96.76%、78.33%，污水垃圾处理能力持续提高。

　　（五）着力改革创新，动力活力不断迸发。围绕破解经济社会深层次矛盾和问题，深入推进基础性关键性改革和创新创业工作。2014—2017年，确定的566项重点改革事项已基本完成，2018年的改革事项正在有序推进，其中商务综合行政监管执法体制改革、地方立法、国企改革、食品安全风险防控、不动产登记等改革事项走在全省乃至全国前列。创新型城市建设成效突出，2014年以来荣获国家、省部级科技进步奖17项，光分路器芯片项目荣获国家科技进步二等奖，国立光电公司连续两年荣获国家创新创业大赛一等奖，目前已建成6个国家和省级创新园区，与全国70余所高等院校、科研院所合作建成88家省级以上创新平台，科技进步贡献率提高到57%，被确定为全国创新驱动示范市、中组部人才工作联系点、全国返乡农民工创业创新示范基地等，获批创建全国金属镁产品质量提升示范区。

　　（六）着力民生福祉，社会大局保持稳定。始终坚持以人民为中心的发展思想，每年都拿出八成左右的财力为群众办一批实事好事，人民群众获得感、幸福感、安全感不断增强。公共就业创业服务体系和政策体系不断完善，累计新增城镇就业16.9万人、农村劳动力转移就业9.2万人，带动居民人均可支配收入由15754元提高到22262元。城乡居民医保低保、城镇居民和职工医保支付比例、城乡居民养老保险金、企业退休人员基本养老金、特困人员供养、基本公共卫生服务经费、大病保险筹资等标准持续提高。以提高质量和促进公平为重点，有序推进教育资源扩容提质和均衡发展，学校数量和办学质量稳步提高，本科教育实现零的突破。加快构建覆盖城乡的医疗服务体系，三甲医院创建成功，被确定为第四批城市公立医院改革国家联系试点。大力推动文化发展繁荣，民俗文化节、樱花文化节、淇河诗歌文化系列活动、文博会、食博会、镁博会、环淇河自行车赛等规模档次和品牌影响持续提升，大运河浚县段、黎阳仓遗址入选世界文化遗产名录。扎实推进平安建设，安全生产、信访稳定、社会治安形势保持平稳，应急管理工作得到加强，非法集资、问题楼盘、拖欠农民工工资等突出问题得到有效防控。

　　四年多来，我们切实加强政府自身建设，坚决贯彻党要管党、全面从严治党方针，认真开展党的群众路线教育实践活动、“三严三实”专题教育、“两学一做”学习教育等，不断强化行政监察、审计监督，着力整治懒政怠政、为官不为行为，风清气正、干事创业的氛围更加浓厚。我们切实加强民主法治建设，自觉接受人大法律和工作监督、政协民主监督、社会监督，认真办理人大代表建议和政协提案，公众法治环境满意度全省领先，被表彰为全国法治宣传教育先进城市、全国法治城市创建工作先进单位，在多次获评全省依法行政责任目标考核优秀等次的基础上，今年被省政府表彰为依法行政工作先进省辖市。

　　我们切实加强精神文明建设，扎实推进社会主义核心价值体系学习教育，认真落实意识形态领域工作责任制，广泛开展各类精神文明创建活动，涌现出了一批国家、省先进典型人物，“鹤壁好人”精神更加凸显。我们切实加强国防教育、国防后备力量及人民防空建设，支持军队改革发展，推进军民融合深度发展，驻鹤人民解放军、武警部队和民兵预备役为地方发展和安全稳定作出了重要贡献，连续五届被评为省双拥模范城。妇女儿童、残疾人、红十字、慈善等事业健康发展。民族、宗教、外事、侨务、对台、统计、档案、史志、气象、地震、邮政、援疆等工作取得了新的成绩。

　　更加值得回顾总结的是，全市人民围绕落实“四个着力”、打好“四张牌”、让中原更加出彩，团结一心、开拓奋进，展现了心齐、气顺、风正、劲足的良好精神状态，做了许多打基础利长远、提士气扩影响的工作，很多方面取得了出彩业绩。在发展思路方面，市九次党代会以及市委九届四次、五次、六次全会胜利召开，进一步明确了我市的战略定位、奋斗目标、基本要求、重点任务等，为今后一个时期的发展指明了方向、提供了遵循、绘就了蓝图。在转型发展方面，因调整改造老工业基地力度较大，培育新产业新业态新模式、承接产业转移和产业合作等工作成效突出，今年我市受到了国务院和省政府的通报表彰，并被赋予一系列含金量较高的配套激励措施，创建产业转型升级示范区也得到国家和省里的明确支持。在现代农业方面，去年在首批全国农业农村信息化示范基地评估考核中排名第一，整市建制国家现代农业示范区进入基本实现阶段，农业现代化综合指数全省第一，农业标准化、信息化、科技化以及粮食高产创建等继续保持全国领先。在城乡建设方面，我市成功入选第三批国家新型城镇化综合试点，跻身中原城市群核心发展区，成为全国首家国土空间优化发展实验区并印发全国首个市级层面的开发格局纲要，与城市总体规划、“十三五”规划等实现了多规合一”。在社会治理方面，“一评四会”基层矛盾纠纷预防化解机制在全省推广，连续17年保持“六个不发生”，连续3届获评全国社会治安综合治理优秀市，成功夺取全国综治工作最高奖项长安杯”，《河南社会治理发展报告2018）》显示，我市居民幸福感位居全省第二。在生态建设方面，成功创建国家森林城市、国家卫生城市、国家园林城市，省级文明城市获得提名，我市在2017—2018年秋冬季“2+26”城市空气质量考核中获优秀等次第七名、省通道城市排名第一，被九三学社中央确定为新农村建设农业面源污染治理示范点。在试点示范方面，我市的现代农业、循环经济、质量强市等超过50项工作被列入国家级试点示范，仅海绵城市、节能减排、清洁取暖就将获43.8亿元中央、省财政补助，拉动社会投资200亿元左右，其中节能减排连续三年考核优秀，清洁取暖首年度考核全国排名第一。

　　以上成绩，是以习近平同志为核心的党中央坚强领导的结果，是省委、省政府和市委科学施策的结果，是市人大和市政协大力支持的结果，更是全市人民拼搏奉献的结果。在此，我代表市人民政府，向全市人民，向各民主党派、各人民团体和各界人士致以崇高的敬意，向关心支持鹤壁发展的各界朋友表示衷心的感谢！

　　成绩来之不易，经验弥足珍贵，应当倍加珍惜、传承发扬。主要是：坚持党对一切工作的领导，以习近平新时代中国特色社会主义思想为指导，着力强化党的建设，统筹推进“五位一体”总体布局和协调推进“四个全面”战略布局，坚持“四个着力”，打好“四张牌”。坚持以人民为中心的发展思想，牢记全心全意为人民服务根本宗旨，把更好满足人民日益增长的美好生活需要作为发展的根本目的，一切依靠人民，一切为了人民，始终在“七个有所”上求提升。坚持贯彻新发展理念和高质量发展要求，牢记发展第一要务，坚定不移贯彻创新、协调、绿色、开放、共享的发展理念，大力推动高质量发展和供给侧结构性改革，持续推进质量变革、效率变革、动力变革。坚持稳中求进工作总基调和转型创新、提升求进工作指向，尊重发展规律，加快转变经济发展方式、优化经济结构、转换增长动力，实现经济平稳增长、质量效益提高互促共进。坚持落实1555”发展思路，这是市委九届四次全会在以往我市发展思路基础上的传承和创新，符合上级精神和我市实际，是全市发展的任务书和路线图。

　　同时，我们也清醒地认识到，当前经济社会发展还面临不少困难和挑战。发展不平衡不充分，经济下行压力依然较大，科技创新和转化能力不强，要素供给更加紧张，部分企业生产经营困难；城乡之间、新老城区之间发展水平、功能面貌差距依然较大，三次产业结构不优，服务业短板依然突出；质量效益有待提高，企业效益、财税收入和居民增收动力不足；利益群体和诉求更趋多元复杂，维护社会稳定压力仍然较大；统筹转型发展与精准脱贫、污染防治做得不够到位，民生保障改善依然任重道远；政府职能转变和机构设置还不到位，一些政府工作人员能力素质不适应新时代要求，一些领域不正之风和腐败问题不容忽视。对此，我们一定勇于担当，着力解决，决不辜负全市人民的期望和重托。

　　二、今后五年的目标任务

　　今后五年，是我市率先全面小康、建设品质“三城”的关键时期。当前，世界经济仍处于深度调整期，虽然存在保护主义加剧、地缘政治风险上升等不稳定不确定因素，但有望继续复苏；我国经济由高速增长阶段转向高质量发展阶段，虽然需要应对可以预料和难以预料的风险挑战，但总体运行稳中有进，特别是国家坚持新发展理念，大力推动高质量发展，正在加快建设现代化经济体系，一系列宏观政策效应持续释放；省里聚焦聚力“三区一群”建设，综合优势持续巩固，战略和政策叠加效应更加凸显。综合来看，我市面临着难得的历史机遇和诸多的有利条件，发展长期向好的基本面没有变，仍处于大有作为的战略机遇期。

　　在今后的工作中，我们将高举习近平新时代中国特色社会主义思想伟大旗帜，深入学习贯彻党的十九大精神和习近平总书记调研指导河南时的重要讲话精神，全面落实省委、省政府和市委各项决策部署，紧扣我国社会主要矛盾变化，落实新发展理念和高质量发展要求，坚持稳中求进工作总基调和转型创新、提升求进工作指向，统筹推进“五位一体”总体布局和协调推进“四个全面”战略布局，着力打赢打好“三大攻坚战”、推进转型发展、促进经济社会持续健康发展，努力谱写新时代品质“三城”建设新篇章，奋力在中原更加出彩中走在前、出重彩。

　　根据市委九届四次全会提出的分阶段目标，结合当前我市发展实际和面临形势，建议今后五年经济社会发展的主要目标：一是速度质量更加优化，主要经济指标年均增速高于全省平均水平，城乡居民收入与地区生产总值增长同步，人均地区生产总值和城乡居民收入走在全省前列。二是转型步伐更加坚实，产业高端化、绿色化、智能化、融合化水平显著提高，科技实力和创新能力大幅跃升，先进制造业强市、特色服务业名市、创新型城市建设初见成效。三是城乡发展更加协调，新型城镇化和城乡一体化质量明显提升，乡村振兴和区域协调发展取得重要进展，农业现代化保持全国领先，城乡基础设施和公共服务体系更加健全均衡。四是生态文明更加向好，绿色、低碳、循环、海绵、节约城市形成品牌，环境指标保持全省前列，文明城市创建取得重大进展，社会文明程度显著提高。五是人民生活更加幸福，现行标准下农村贫困人口全部脱贫，公共事业全面进步，社会保障和社会治理体系更加完善高效，社会大局保持和谐稳定，居民生活条件和生活质量得到明显改善。

　　工作中需要把握好以下几个原则：一是坚持“一个指引”，就是坚持党对一切工作的领导，牢固树立“四个意识”，自觉做到“四个服从”，坚决维护习近平总书记党中央的核心、全党的核心地位，坚决维护党中央权威和集中统一领导，不折不扣贯彻落实党中央的决策部署。二是坚持“三个基本”，就是坚持全面贯彻党的基本理论、基本路线、基本方略，认真践行以人民为中心的发展思想和新发展理念，落实稳中求进工作总基调和高质量发展要求，推进全面依法治市和全面从严治党，以更高站位、更宽视野推进改革开放。三是坚持“五个导向”，就是以提升求进的劲头推动各项事业全方位高质量进步，以创新发展的理念激发全社会创造活力，以示范引领的意识做精品、创品牌、成特色、树标杆，以厚植优势的思维持续增强综合竞争力，以勇争一流的追求创造出彩业绩。四是坚持“五个举措”，就是强化改革推动、开放带动、项目拉动、“双创”驱动、环境促动，以深化改革破难题增红利，以扩大开放应多变求多效，以项目建设拉投资强后劲，以创业创新激活力添动力，以优化环境提形象促集聚。五是坚持“五个协调”，就是推动改革发展稳定协调统一、速度质量效益协调统一、经济社会生态协调统一、产业城市乡村协调统一、重点主线全面协调统一，扎实推进率先全面小康、建设品质“三城”各项工作。

　　三、2018年政府工作安排

　　今年是贯彻党的十九大精神的开局之年，是改革开放40周年，是实施“十三五”规划承上启下的关键一年，也是我市决胜率先全面小康、开启新时代品质“三城”建设新征程的重要一年，还是新一届政府的起步之年。建议全市经济社会发展主要预期目标为：地区生产总值增长8%左右、在实际工作中争取更高质量和更好效益，其中第三产业增加值增长9.5%左右；规模以上工业增加值增长8.5%左右；一般公共预算收入增长8%左右；居民人均可支配收入增长8%以上；固定资产投资增长10%左右；社会消费品零售总额增长12%左右；城镇新增就业2.7万人以上；常住人口城镇化率提高1.2个百分点以上；居民消费价格上涨3%左右；节能减排、环境保护等约束性指标完成省下达任务。

　　为实现以上目标，我们重点抓好以下8个方面的工作：

　　（一）打赢打好“三大攻坚战”，夯实率先全面小康基础。中央提出的防范化解重大风险、精准脱贫、污染防治“三大攻坚战”，事关全面小康目标的实现，是重中之重的政治任务，必须坚决打赢打好。防范化解重大风险。健全风险防控机制，着力解决突出问题，牢牢守住不发生区域性系统性风险的底线。加强企业风险排查和分类管控，及时帮助企业解决实际困难；实施企业家素质、企业成长和创新平台培育提升工程，引导企业聚焦主业、适度规模、规范治理、注重研发、提升品牌，增强高质量发展内生动力和适应新常态下市场经济能力；深化金融与实体经济良性互动，做好省普惠金融试点市建设工作，提高银行业支持实体经济发展能力和企业融资能力，促进企业稳健有序发展。科学制定政府债务风险化解实施方案，确保年底前存量债务化解迈出实质性步伐、退出风险提示区，坚决杜绝各类违法违规举债行为、严控债务增量。深入开展非法集资处置、问题楼盘化解、不良贷款盘活清收等工作，依法打击恶意逃废银行债务行为，加快社会信用体系建设，着力维护金融秩序、优化金融环境。深入推进精准脱贫。坚持把提高脱贫质量放在首位，聚焦深度贫困乡村和特殊贫困群体“两大重点”，打好产业扶贫、就业创业扶贫、生态扶贫、金融扶贫“四大硬仗”，开展健康扶贫、教育扶贫、扶贫助残、易地扶贫搬迁、危房改造清零、扶贫扶志“六大行动”，实施交通扶贫、饮水安全、电网升级、环境整治“四大工程”，细化完善政策体系，加快构建专项扶贫、行业扶贫、社会扶贫“三位一体”大扶贫格局，注重将扶贫同扶志、扶智更好结合起来，进一步强化资金保障、政策保障、队伍保障、机制制度保障和作风纪律保障，确保18个贫困村脱贫摘帽、7875名农村贫困人口稳定脱贫。做好台前县扶贫对口帮扶工作。扩大污染防治成效。牢固树立绿水青山就是金山银山的理念。深入推进大气污染防治，确保空气优良天数220天以上、可吸入性颗粒物（PM10）平均浓度每立方米110微克以下、细颗粒物（PM2.5）平均浓度每立方米61微克以下，力争全年煤炭消费总量下降5%。持续实施水污染防治，确保河长制相关要求全面落实、淇河流域生态质量持续改善、市域河流断面各项指标稳定达标、城市建成区内黑臭水体全部消除，加强饮用水水源和地下水环境保护，积极争创国家水生态文明试点城市和省级节水型城市。积极开展土壤污染防治，完成农用地土壤污染状况详查和建设用地土壤污染地块名录，有效防范新增土壤污染。加强生态保护修复，做好绿化造林、静脉产业园建设和节能减排、循环经济、海绵城市、清洁取暖等试点建设，高水平建设绿色、低碳、循环、海绵、节约城市。

　　（二）激发市场主体活力，促进经济持续健康发展。大力发展实体经济，持续稳增长保态势，努力打造发展新优势。扩大有效投资。持续优化投资环境，激发民间投资主体活力，扩大投资规模，改善投资结构，提高投资效益。抓好100个重点项目建设，力争全年谋划重大项目60个以上、新开工50个、竣工11个，完成投资340亿元以上。优化营商环境。聚焦非公有制经济高质量发展，以更加鲜明的导向、更加坚定的信心、更加务实的举措，营造非公有制经济健康发展的浓厚氛围，促进非公有制经济创新发展、转型发展、提质发展。推进上市挂牌。建立健全促进企业上市的工作机制，强化上市后备企业培育，完善落实奖励扶持政策，着力提升企业资本市场融资能力。夯实载体平台。推进产业集聚区提质发展和服务业“两区”提速扩容，力争宝山循环经济产业集聚区晋升二星级，全面提升商务中心区和特色商业区发展水平，增强区域影响力和辐射力，服务业“两区”完成固定资产投资50亿元以上。推动质量提升。扎实做好质量强市建设，继续实施增品种、提品质、创品牌专项行动，弘扬“工匠精神”，引导企业开展对标达标活动。培育消费热点。深入挖掘健康、养老、教育、体育、文化、旅游等消费领域潜力，谋划推进健身休闲产业发展，鼓励引导绿色产品消费，大力发展共享经济。

　　（三）深入推进转型发展，不断提高供给质量效益。抓住国家实施新一轮老工业基地振兴战略的机遇，用好国家、省支持我市创建产业转型升级示范区的政策，紧抓推动经济高质量发展的着力点增长点，大力发展互联网、大数据、云计算、人工智能等新兴产业，培育更多的增长因素、增长业态、增长动力，加快调整产能、产业、产品结构，助推经济结构战略性转型。深化供给侧结构性改革。坚决破除无效供给，再退出煤炭产能36万吨，依法依规推进停滞半停滞项目和企业、闲置厂房楼宇盘活工作。积极培育新动能，严格执行环保、能耗、质量、安全等标准，推动工业企业绿色化、智能化、技术化改造。切实降低实体经济成本，落实降低税收负担政策，继续清理整治涉企收费、乱收费行为，降低制度性交易成本和生产经营成本。建设先进制造业强市。出台实施《中国制造2025鹤壁行动纲要》和《2018年工业“3+2”产业推进方案》，加快打造清洁能源与新材料、绿色食品两个千亿级产业集群，汽车零部件与电子电器超五百亿级产业集群，镁精深加工、现代家居两个超百亿级产业集群。建设特色服务业名市。深入实施“2+3”现代服务业发展提升行动计划，加快打造南太行重要旅游集散地和国家全域旅游示范区、华夏文明传承保护创新区、国内中高端养生养老基地、豫北重要物流节点城市、网络经济活跃区。改造提升商贸流通等传统服务业，积极发展现代金融、商务会展等新业态新模式，推动生产性服务业向专业化和价值链高端延伸、生活性服务业向精细化品质化提升。保持现代农业全国领先地位。全面提升11个省级农业产业化集群和22个市级现代农业园区发展水平，大力发展优质高效作物，持续推进一二三产业融合发展，不断提高农业发展质量效益和产业竞争力。抓好全国畜牧业绿色发展示范市、全国粮油绿色高产高效示范基地建设，积极争创生态原产地产品保护示范城市。大力培育新型经营主体，完善现代农业产业体系、生产体系、经营体系。全面落实粮食安全责任制，持续提升粮食安全保障水平。

　　（四）推动城乡融合发展，持续优化区域发展格局。突出全域统筹、协同联动，健全城乡融合发展体制机制，进一步提升城乡环境质量和人民生活质量。科学推进新型城镇化。提高核心区首位度，高标准建设淇滨东部片区，进一步加快淇滨区、开发区、城乡一体化示范区相关设施的共建共享；加快推进鹤淇一体化，促进淇县通过鹤淇产业集聚区的带动向北发展。提速城市组团建设，加快实施鹤山区独立工矿区改造搬迁和山城老工业区搬迁改造工程，支持山城区创建国家城区老工业区搬迁改造示范工程；推动浚县向主城区方向发展，加快白寺物流产业园区建设，有序实施浚县旧城改造和新城开发。提质重点镇发展，加快镇域特色产业发展，培育百强乡镇。深入实施百城建设提质工程。坚持高起点规划、高标准建设、高效率管理、高水平经营，做到硬件与软件、新区与老区、地上与地下、宜业与宜居“四个统一”，做好以绿荫城、以水润城、以文化城、以业兴城“四篇文章”。有序推进城中村、背街小巷和老旧小区改造，抓好交通秩序治乱、市容卫生治脏、生态环境治污、公共服务治差。积极创建省“公交优先”示范城市，加快干线公路和“四好农村路”建设。有序推进智慧城市建设，提高城市精细化、智能化管理水平。大力实施乡村振兴战略。按照产业兴旺、生态宜居、乡风文明、治理有效、生活富裕的总要求，加快编制实施乡村振兴战略规划，推动乡村产业振兴、人才振兴、文化振兴、生态振兴、组织振兴。扎实推进农村综合配套改革，全面启动农村集体产权制度改革，加快农村集体资产清产核资。进一步规范土地流转，推动小农户和现代农业发展有机衔接，促进小农户家庭经营为基础与多种形式适度规模经营为引领相协调。强化农村基层组织建设，打造懂农业、爱农村、爱农民的“三农”工作队伍。深入开展农村人居环境整治行动，加快打造生态宜居美丽乡村。加大传统村落、传统民居和历史文化名村名镇保护力度。提升城乡融合发展水平。发挥城乡一体化试点效应，强化“一基本两牵动三保障”，加快制定“人地”“人钱”挂钩配套举措，深化户籍制度、居住证等改革，将住房保障等纳入居住证基本公共服务范围，让符合条件的农业转移人口在城市落户定居，进一步提高户籍人口城镇化率。

　　（五）深化改革开放创新，加快发展动能接续转换。坚持把改革开放创新作为推动发展的动力源泉，不断提升改革成效、开放层次和创新质量。全面深化改革。深化“一网通办”前提下的“最多跑一次”改革，推进审批服务便民化，进一步简政放权、创新监管、优化服务，推动政府治理体系和治理能力现代化。深化土地综合改革，做好城镇规划建设用地、农村耕地和乡村建设用地改革工作，加大违法违规用地、低效利用土地处置力度，提高已批土地供地率，增强土地支撑保障作用。深化国有企业改革，推进企业产权结构、组织结构、治理结构改革，全面完成浚县农场剥离企业办社会职能工作。深化财税体制改革，加强财源建设，完善综合治税体系，稳妥推进市以下财政事权与支出责任划分改革试点，合规合理划分财权事权，认真落实费改税相关政策。深化投融资体制改革，规范政府投资行为，强化企业投资主体地位，放宽放活社会投资。统筹做好其他方面的改革。持续扩大开放。深度融入国家“一带一路”、京津冀协同发展战略和省“三区一群”建设规划，务实推进豫北区域协同发展，积极承接北京非首都功能疏解和长三角、珠三角等重点区域、重点城市的产业转移。加快对接融入河南自贸区，推进鹤壁综合保税区和跨境电子商务试验区建设，完善外贸发展政策措施，提高通关便利化水平。紧盯央企名企和今年确定的100个重点招商项目，坚持引资金与引技术、引平台、引人才并重，吸引国内外知名企业和机构在鹤壁集聚发展。强化创新驱动。坚持以科技创新为核心，率先复制享受郑洛新国家自主创新示范区等先行先试政策，积极争创国家可持续发展议程创新示范区和国家农业产业技术示范区，加快建设创新型城市。创新体制机制，完善政策措施，深入实施“四个一批”行动计划，新发展一批创新引领型企业、建设一批创新引领型平台、培育一批创新引领型人才、引进一批创新引领型机构。推动科技金融深度融合，做好国家知识产权质押融资和省科技与金融结合试点城市创建工作。完善创新投入体系，着力提高全社会研发投入占生产总值的比重和财政科技投入占公共财政支出的比重。

　　（六）协同发展社会事业，全面提升公共服务水平。坚持抓重点、补短板、强弱项，着力优化基本公共服务供给。发展公平而有质量的教育。实施好第三期学前教育行动计划，改扩建公办幼儿园6所。提高城乡义务教育一体化发展水平，探索推进中小学集团化办学模式，优化教育资源配置和布局，改造农村薄弱学校7所，建成投用中小学3所，着力解决中小学生课外负担重、择校热、大班额等突出问题。以经济社会发展需求为导向，引进扩充优质职业教育资源，多措并举提高职业教育办学成效。推进普及高中阶段教育，继续抓好民办教育、特殊教育、继续教育、网络教育、家庭教育。健全学生资助制度，实现建档立卡贫困家庭学生资助全覆盖，让每个孩子都有平等机会通过教育改变命运、成就梦想。提升健康服务水平。健全医疗卫生服务体系，做好城市公立医院综合改革、医疗医保医药联动改革，加快分级诊疗、家庭医生签约服务和医联体建设步伐。推进基本公共卫生服务均等化，提高城乡居民基本医疗保险人均财政补助标准，加强重大疾病防控和妇女儿童、老年人、残疾人等重点人群健康服务，免费开展出生缺陷产前筛查和新生儿疾病筛查，完成1.7万名农村适龄、城市低保适龄妇女“两癌”免费筛查。巩固扩大国家卫生城市创建成果。加强和创新食品药品监管，做好国家食品安全城市创建工作，让消费者买得安心、吃得放心。广泛开展全民健身活动，加快完善县区“两场三馆”和各类体育健身设施。建设文化强市和文明城市。坚定文化自信，培育和践行社会主义核心价值观，持续加强意识形态工作，推进群众性精神文明创建，开展公民思想道德素质、诚信守法、文明风尚提升行动，叫响“鹤壁好人”品牌。大力发展文化事业文化产业，深入实施“五个一工程”和文化惠民工程，繁荣文艺创作，持续提升文化节会层次和水平，加快建设国家级公共文化服务体系示范项目。加强文物保护利用和文化遗产保护传承，讲好鹤壁故事，积极建设淇河文化经济带和大运河文化带。继续做好文化体制改革、新型智库建设、网络建设治理、志愿服务、扫黄打非等工作。

　　（七）切实保障改善民生，着力增进人民群众福祉。坚持在发展基础上多办利民实事、多解民生难事，不断提升人民群众的获得感、幸福感、安全感。持续增加居民收入。拓宽居民劳动收入和财产性收入渠道，健全收入分配机制，合理调整社会最低工资标准，保持居民收入与经济同步增长、劳动报酬与劳动生产率同步提高，努力实现城乡居民收入增速走在全省前列。稳定扩大社会就业。加强全方位公共就业服务，支持以创业带动就业，促进高校毕业生、就业困难人员、退役军人等重点群体多渠道就业创业，推动农村劳动力转移就业，妥善做好过剩产能化解、“僵尸企业”处置等过程中下岗分流职工再就业工作，着力扩大各类人员返乡下乡创业成效。健全劳动关系协商机制，构建和谐劳动关系。依法治理拖欠农民工工资问题。加大住房保障力度。加快建立多主体供给、多渠道保障、租购并举的住房制度。强化公租房保障，逐步将符合条件的机关事业单位、高等院校、公立医院、科研院所和国有企业青年人才，以及其他新就业无房职工、外来务工人员纳入保障范围。紧抓国家启动新的三年棚改攻坚计划，在做好棚改安置房逾期未交付使用问题整改的同时，新开工棚改安置住房1784套，基本建成各类保障性住房12339套，完成农村危房改造300户。坚持房子是用来住的、不是用来炒的定位，维护房地产市场健康发展。强化民生保障兜底。加快多层次社会保障体系建设，全面实施全民参保计划。积极参与养老保险全国统筹和工伤保险基金省级统筹，完成机关事业单位养老保险改革，提高退休人员基本养老金和城乡居民基础养老金。完善城乡居民基本医疗保险制度、大病保险、困难群众大病补充医疗保险和医疗救助制度，提高农村贫困人口门诊慢性病和门诊重特大疾病保障水平。稳步提高城乡低保、特困人员供养、抚恤优待等标准，全面落实困难残疾人生活补贴和重度残疾人护理补贴政策，健全农村留守儿童、妇女、老人关爱服务体系和社会救助体系。大力发展慈善公益事业，让每一个身处困境者都能得到社会的关爱和温暖。推动社会治理创新。全面落实安全生产责任制，强化重点行业领域安全专项治理，实现“一个杜绝、三个持续下降”目标。完善社会矛盾纠纷多元化解机制，创新信访工作方式，依法及时解决群众合理诉求。加强社会治安防控体系建设，坚决依法打击各类违法犯罪和暴力恐怖活动，深入开展扫黑除恶专项斗争。做好社区物业服务、困难群体法律援助、防灾减灾救灾等工作，维护社会大局和谐稳定。

　　加强国防教育、国防动员和国防后备力量建设，支持国防和军队改革，支持驻鹤解放军、武警部队、民兵预备役和人民防空建设，深入开展双拥共建，巩固军政军民团结，推进军民融合深度发展。全面贯彻党的民族政策和宗教工作基本方略，促进民族团结、宗教和睦。支持工会、共青团、妇联、工商联等人民团体更好发挥桥梁纽带作用。做好外事、侨务、对台、审计、统计、档案、史志、气象、地震、邮政、援疆等工作。

　　（八）不断加强自身建设，有效提升政府治理能力。顺应人民群众新期待，适应实践发展新要求，努力造就信念过硬、政治过硬、责任过硬、能力过硬、作风过硬的干部队伍，加快建设人民满意的政府。加强政治建设。将政治建设作为政府自身建设的根本要求，把维护以习近平同志为核心的党中央权威和集中统一领导作为严格政治准则和根本政治要求，在政治立场、政治方向、政治原则、政治道路上，始终同以习近平同志为核心的党中央保持高度一致。高标准严要求推进中央巡视反馈意见整改工作，扎实做好巡视“后半篇文章”。坚持依法行政。全面推进依宪施政、依法行政，坚持科学民主依法决策和严格规范公正文明执法，全面推进政务公开和法律顾问制度，加快建设法治政府。依法接受人大及其常委会的法律和工作监督，自觉接受人民政协的民主监督，主动接受社会监督，认真办理人大代表建议和政协提案，深入听取民主党派、工商联、无党派人士和各人民团体的意见。保持勤政廉洁。扎实推进“两学一做”学习教育常态化制度化，认真开展“不忘初心、牢记使命”主题教育。坚决贯彻落实中央八项规定及实施细则精神，驰而不息纠正“四风”，严肃查处懒政怠政、为官不为行为。落实全面从严治党主体责任和“一岗双责”，坚决查处腐败和损害群众利益的行为，严肃对待、认真解决群众反映强烈的问题。提高政府效能。优化政府机构设置和职能配置，加快形成职责明确、依法行政的治理体系。增强“八种本领”和责任担当意识，强化容错免责和失职追责，大兴学习之风、调查研究之风、干事创业之风，务求工作定一件干一件、干一件成一件，提高政府执行力和公信力。

　　各位代表，幸福是奋斗出来的，只有凝聚力量、拼搏实干，才能达到预期目标、成就美好未来。让我们更加紧密地团结在以习近平同志为核心的党中央周围，在省委、省政府和市委的坚强领导下，咬定目标、砥砺前行，合力开创鹤壁更加美好的明天！
